# Decision fatigue among military primary care physicians: a retrospective study

**DOI:** 10.1186/s13584-026-00757-0

**Published:** 2026-05-13

**Authors:** Shir Gutman, Dana Eskenazi Anoshi, Hadasa Sharshevsky, Barak Gordon

**Affiliations:** 1 Faculty of Medicine, The Hebrew University of Jerusalem, Jerusalem, Israel; 2 Department of Dermatology, Tel Aviv Sourasky Medical Center, Tel Aviv, Israel; 3 Department of Biostatistics, Medical Corps, Israel Defense Forces, Ramat Gan, Israel; 4 Department of Military Medicine, Faculty of Medicine, The Hebrew University of Jerusalem, Jerusalem, Israel; 5 Medical Corps, Israel Defense Forces, Ramat Gan, Israel

**Keywords:** Decision fatigue, Military physicians, Clinical decision-making, Workday trends

## Abstract

**Background:**

Decision fatigue, based on Baumeister’s “Strength Model of Self-Control”, suggests that the quality of decision-making declines with the accumulation of decisions over time. This phenomenon, previously observed in various professional fields, may impact military physicians who make critical and organizational decisions. We aimed to assess how decision-making changes throughout the workday among military physicians in a clinic setting.

**Methods:**

A retrospective observational analysis was conducted using a database of 2 million medical encounters from the military medical record system between 2015 and 2019. Decision outcomes were tracked for exemptions from specific duties granted, sick leave issued, days of sick leave, emergency department referrals, antibiotic prescriptions for upper respiratory tract infections and spinal X-ray referrals for low back pain – in accordance with appointment time and physician employment type.

**Results:**

Findings showed a decrease in specific-duty exemptions granted by physicians until lunchtime, a peak post-lunch and a subsequent decline in the afternoon (*p* < 0.001). Referrals to the emergency department increased through the morning, dipped post-lunch and rose again by day’s end (*p* < 0.001). Sick leave issuance and the number of days granted both declined as the day progressed, with the steepest decline in the afternoon (*p* < 0.001). Rates of antibiotic prescription for URTIs and spinal X-ray referrals for LBP did not vary significantly throughout the day. Civilian physicians tended to issue more sick leave, exemptions and referrals than regular duty physicians, though both groups demonstrated similar trends throughout the day.

**Conclusions:**

This study highlights the influence of decision fatigue on clinical decision-making among military physicians, showing a time-based decline in granting sick leave and specific-duty exemptions, with an increase in emergency referrals towards the end of the workday. These might have system-wide implications such as overutilization of healthcare, and increased health expenditure. Recognizing and addressing decision fatigue is important to mitigate its effects.

## Background

Decision fatigue, a concept rooted in Baumeister’s Strength Model of Self-Control, refers to the decline in self-regulatory capacity due to repeated acts of decision making, a process reliant on finite cognitive resources [1, 2]. The effects of decision fatigue have been empirically demonstrated across diverse fields, where prolonged cognitive demands lead to declining decision quality. Notably, Danzinger et al. found in a study of Israeli parole board decisions that candidates reviewed earlier in the day had significantly higher chances of receiving favorable outcomes, with performance recovering to early morning rates after a lunch break [3].

In healthcare, decision fatigue poses unique risks, as the quality of each decision has direct implications for patient outcomes. Research suggests that as healthcare shifts progress, clinicians may increasingly rely on convenient yet suboptimal choices, often to expedite patient interactions and reduce cognitive strain. For instance, Linder et al. observed that primary care physicians were more likely to prescribe antibiotics as the shift advanced [4]. The assumption is that decision fatigue manifests as an increase in antibiotic prescriptions throughout the day, as this is considered an easier treatment option, allowing for patient satisfaction, short medical encounters and reduced cognitive effort. Similarly, Philpot et al. found an increase in opioid prescriptions for low-back pain by day’s end [5]. Hare et al. found that primary care physicians tended to prescribe clinically appropriate statin therapy less in later appointment hours compared to morning appointments [6]. Oakes et al. commented on a similar tendency among cardiologists [7] Hsiang et al. observed that preventive cancer screening rates dropped as appointments occurred later in the day [8], while Kim et al. reported a steady decrease in flu vaccination rates throughout the day, despite staff instructions to encourage vaccinations [9]. Persson et al. found that decision fatigue among orthopedic surgeons affected surgical scheduling, with patients seen later in the workday 33% less likely to be scheduled for surgery compared to those seen earlier, reflecting the complexity of such decisions [10]. Allan et al. observed that nurses handling medical helpline calls became more conservative in decision making as their shift progressed, with a stronger tendency for emergency referral [11]. Other studies have identified declines in hygiene compliance and preventive screening recommendations [12, 13], indicating that prolonged decision-making tasks lead to compromised patient care practices. Thomspon et al. highlighted that decision fatigue in nursing settings can impair clinical judgment and quality of care [14].

The system-wide implications of decision fatigue have not directly been studied. It is reasonable, though, to presume that systemic tendencies to underperform as the workday or shift advances have economic and epidemiologic impact. For example, reduced statin prescription for secondary prevention of cardiovascular disease might have a downstream effect of increased incidence of cardiovascular events and hospitalizations. Reduced screening for cancer might result in cancer detection at more advanced stages, influencing not only the individual patient but also overall cancer mortality and treatment expenditure.

Interventions such as structured breaks, decision guidelines and workload distribution have been proposed to mitigate these effects. Schmeichel suggests that structured cognitive interventions may help sustain decision quality [15]. Moorhouse recommends reducing decision load through proactive strategies like delegating tasks, using clinical guidelines and incorporating regular breaks [16]. Military primary medicine is a complex microcosmos in regard to decision fatigue. Military primary care physicians face unique cognitive and emotional pressures during the workday, stemming from the complex nature of their decisions. These decisions impact not only the immediate medical, psychological and social well-being of the soldiers they treat, but also the operational capacity and readiness of their units. The value-based conflicts inherent in such decisions often require balancing the principles of beneficence, non-maleficence, patient autonomy and justice. Such demands are likely to exacerbate decision fatigue, potentially leading to a decline in decision quality over the course of the workday. Laour et al. discuss ethical dilemmas in military triage, emphasizing that these challenges can heighten cognitive strain and decision fatigue among military medical personnel [17].

Although previous studies have examined decision fatigue among civilian healthcare providers, research has yet to specifically address military physicians, who operate in an environment that imposes distinct cognitive and ethical challenges. This study aims to examine whether military primary care physicians demonstrate changes in decision-making over the course of the day which may be attributed to decision fatigue. Using retrospective data from the Israeli Defense Forces’ (IDF) Medical Corps computerized patient record (CPR) system from 2015 to 2019, we assessed the impact of cumulative work time on decision endpoints and explored the relationship between physician characteristics, specifically military versus civilian, and this trend.

## Materials and methods

### Study design

This is a data-based retrospective observational study. All data was extracted from the IDF’s CPR system. Data extraction and analysis were conducted while maintaining the confidentiality of identifiable information. Statistical analysis was performed using SPSS version 29. This study was conducted without funding and received exemption approval from the Institutional Ethical Review Committee of the IDF Medical Corps.

### Study population

This study is based on patient encounters between physicians and soldiers in primary care clinics within the IDF Medical Corps from 2015 to 2019. Only encounters conducted by physicians who worked at least 60 days per year and who conducted a minimum of 20 encounters per day were included, ensuring the sample focused on physicians whose primary responsibility was primary care in both rear and combat units. The study period (2015–2019) was chosen to avoid the impact of the COVID-19 pandemic, which presumably altered clinic workflow since February 2020 as well as the Swords of Iron War, beginning October 2023. The study population includes all doctors who treated soldiers in IDF primary care clinics during this period, provided they met the criteria mentioned above. This study population was further divided into subgroups of active-duty military career physicians and civilian physicians employed by the military.

### Variables

Variables were extracted directly from IDF’s CPR system. The primary independent variable was the time of the encounter. For analysis, work hours were divided into hourly intervals, with each encounter assigned a time value based on the hour of occurrence (e.g., encounters from 8:00–9:00 were labeled as 8:00). Additionally, physician type was recorded for each encounter, classifying doctors as either military (active-duty) or civilian employees.

Several dependent variables were analyzed to assess potential decision fatigue among physicians. For encounters where “Upper Respiratory Tract Infection” (URTI) was listed as the diagnosis, it was noted whether an antibiotic was prescribed. For cases of “Lower Back Pain” (LBP), records were checked to see if the patient was referred for X-ray imaging. For each encounter, it was recorded whether an emergency referral was made. Furthermore, occupational medical decisions unique to military medicine were assessed, including whether exemptions from specific tasks (service exemptions) or sick leave were granted, along with the duration of sick leave when applicable. This approach allowed for a comprehensive evaluation of decision-making patterns and potential indicators of decision fatigue.

Indicators such as antibiotic prescriptions for URTIs and imaging referrals for LBP were selected based on prior research demonstrating their sensitivity to decision fatigue [4]. These decisions often involve balancing clinical appropriateness with patient expectations and time pressure, making them ideal for examining changes in decision quality across the workday.

### Statistical methods

The dataset comprised of 2,687,455 encounters from the CPR system spanning 2015 to 2019. Encounters from days when physicians conducted at least 20 appointments between 8:00 and 17:00 were included, resulting in a final sample of 2,012,297 encounters across 69,100 workdays. The sample included 317 physicians, of whom 192 were active-duty military physicians and 125 were civilian physicians. For analysis of “referral for imaging” in cases of lower back pain, a subset of 83,485 encounters with an LBP diagnosis were extracted. Similarly, a subset of 95,809 cases of URTI were analyzed for antibiotic prescription.

Data was analyzed using descriptive statistics, with error bars representing the mean and standard deviation of each variable across work hours. For dichotomous variables, the mean for each hour was calculated. To adjust for individual physician tendencies in decision-making, each variable was normalized by calculating a physician-specific mean and standard deviation across all encounters. Standardized values were then derived for each variable using the following formula, where X represents the value of the dependent variable, $\:\stackrel{-}{x}$ denotes the physician-specific mean of that variable across encounters and s is the physician-specific standard deviation:

$$\:Standardized\:Value=\:\frac{(x-\stackrel{-}{x})}{s}$$

Comparisons between military and civilian physicians were conducted using the chi-square test. To assess changes over the workday, chi-square tests were applied to four time intervals (8–12, 8–13, 12–13, 13–16) for each dichotomous variable, including emergency referral, granting of exemptions, sick leave provision, antibiotic prescription for URTI and imaging referral for LBP. For the continuous variable, “number of sick leave days granted”, comparisons across time intervals were conducted using the Wilcoxon sum rank test. P-values are reported in Table 2 and referenced throughout the results.

## Results

### Distribution of encounters across working hours

**Fig. 1:**
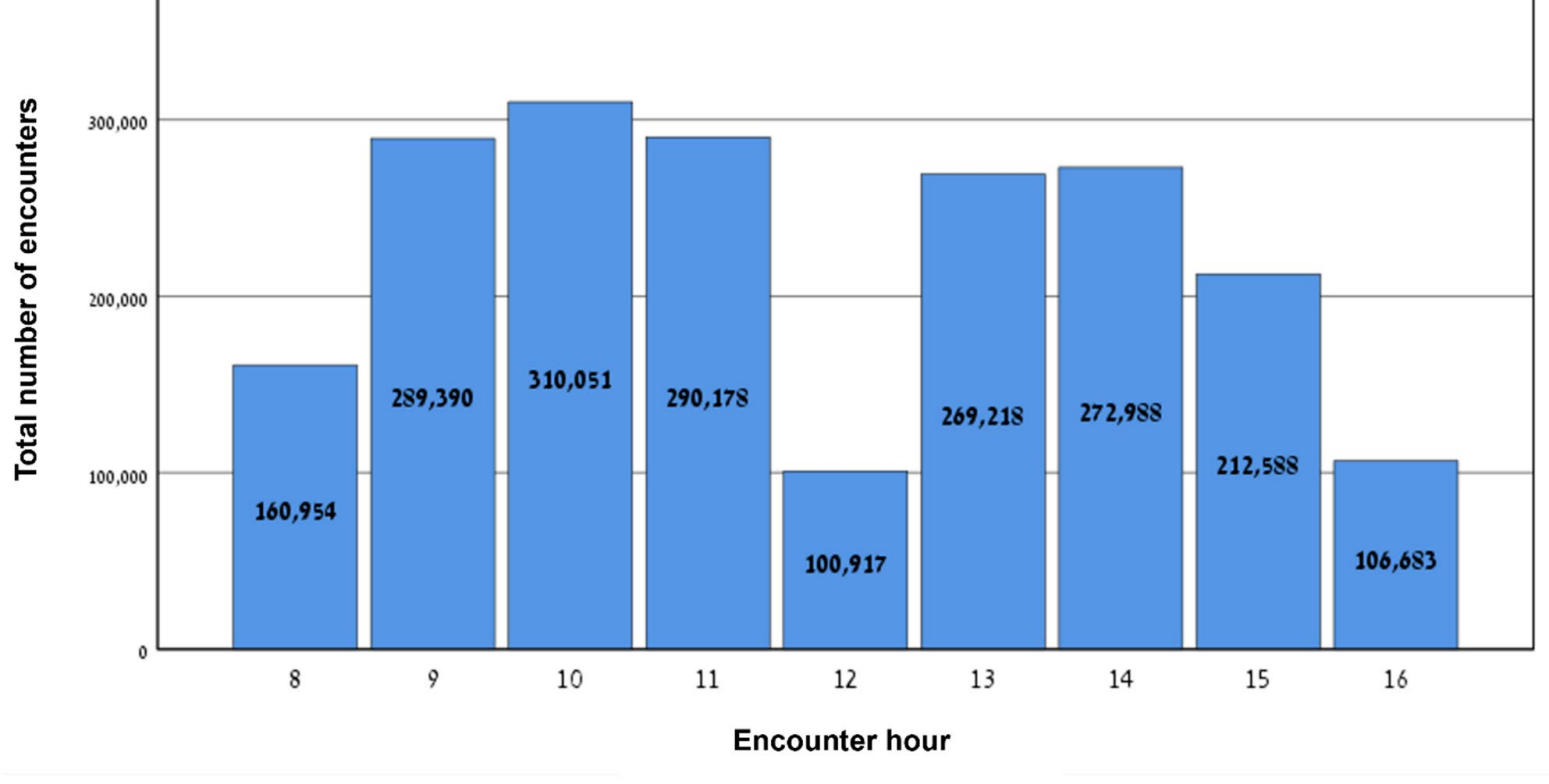
Total number of encounters by hour

An examination of encounters by time of day, presented in Fig. 1, shows a bimodal distribution. There is a relative decrease in encounters during the early morning (8–9), at the end of the afternoon (16–17), and during the presumed lunch break (12–13).

### Relationship between emergency room (ER) referrals and encounter hour

**Fig. 2:**
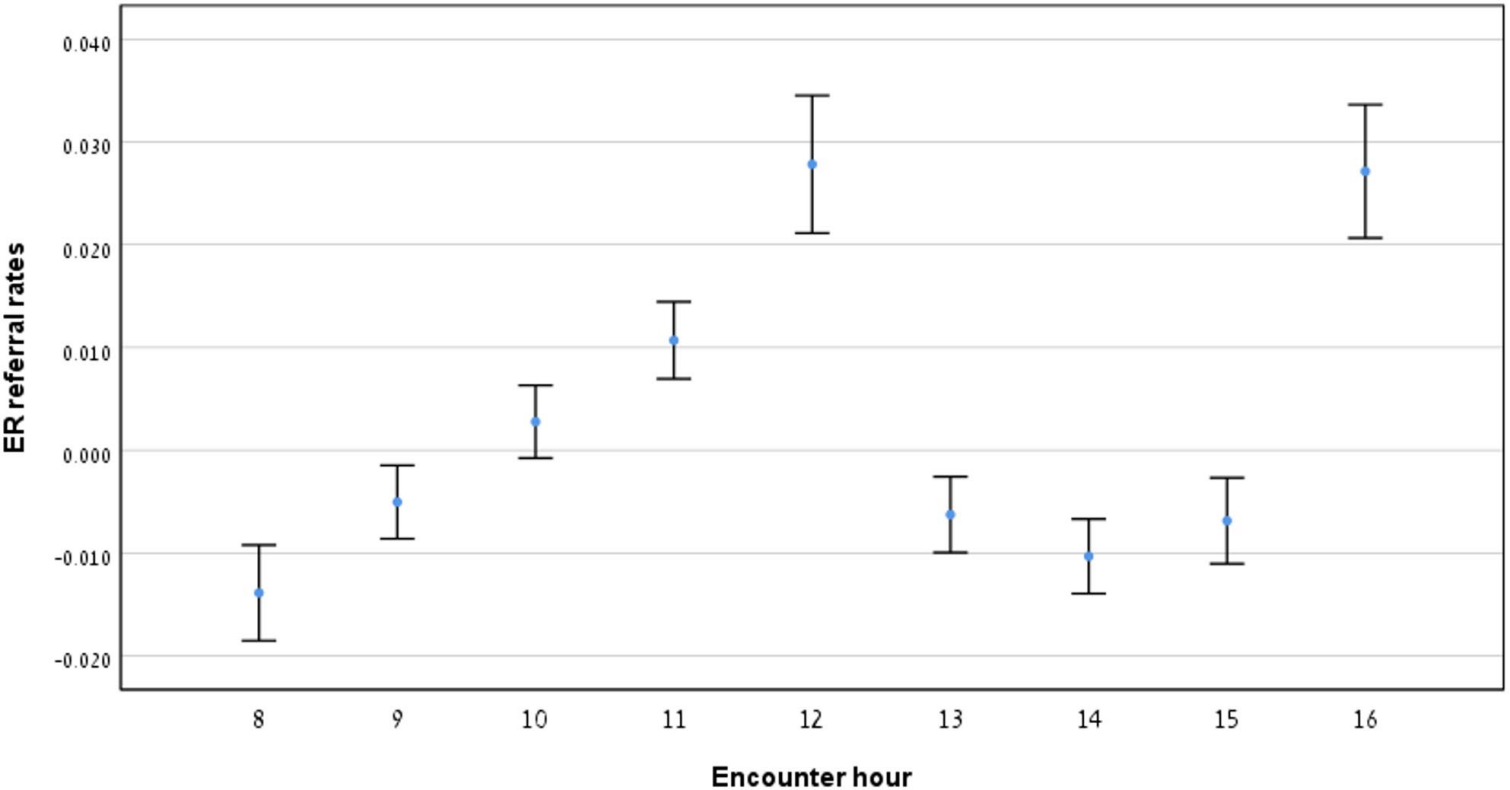
ER referral rates by encounter hour

Fig. 2 displays the proportion of encounters resulting in ER referrals throughout the workday. The proportion of ER referrals increased through the morning, peaked around midday, then decreased slightly in the afternoon before rising again at the end of the workday. Table 1 summarizes encounter data across morning, midday and afternoon hours. No significant difference was found between 8 and 13 (*p* = 0.165, Table 2), while significant increases in ER referrals were observed differences were observed between 8 and 12, 12–13, and 13–16 (*p* < 0.05, Table 2).

**Table 1:** Distribution of key clinical decisions by encounter hour

Hour	ER referrals % (95% CI)	Sick leave % (95% CI)	Service exemptions % (95% CI)	Spinal X-ray referrals for LBP % (95% CI)	Antibiotic prescriptions for URTI % (95% CI)
8	2.3 (2.2, 2.4)	12.4 (12.3, 12.6)	7.8 (7.6, 7.9)	1.6 (1.4, 1.9)	9.7 (9.1, 10.3)
12	3.0 (2.9, 3.1)	10.9 (10.7, 11.0)	5.9 (5.8, 6.1)	1.6 (1.3, 2.0)	9.6 (8.6, 10.5)
13	2.4 (2.3, 2.4)	10.6 (10.5, 10.7)	7.0 (6.9, 7.1)	1.7 (1.5, 1.9)	8.3 (7.8, 8.8)
16	3.0 (2.9, 3.1)	8.0 (7.8, 8.1)	5.6 (5.5, 5.8)	1.4 (1.0, 1.7)	8.8 (8.0, 9.6)

**Table 2:** Statistical comparisons between encounter hours

Comparison	ER referrals	Sick leave	Service exemptions	Spinal X-ray referrals for LBP	Antibiotic prescriptions for URTI
8 vs. 12	< 0.001	< 0.001	< 0.001	0.986	0.812
8 vs. 13	0.165	< 0.001	< 0.001	0.638	0.001
12 vs. 13	< 0.001	0.042	< 0.001	0.709	0.020
13 vs. 16	< 0.001	< 0.001	< 0.001	0.083	0.373
8 vs. 16	< 0.001	< 0.001	< 0.001	0.216	0.084

### Relationship between sick leave issuance and encounter hour

**Fig. 3:**
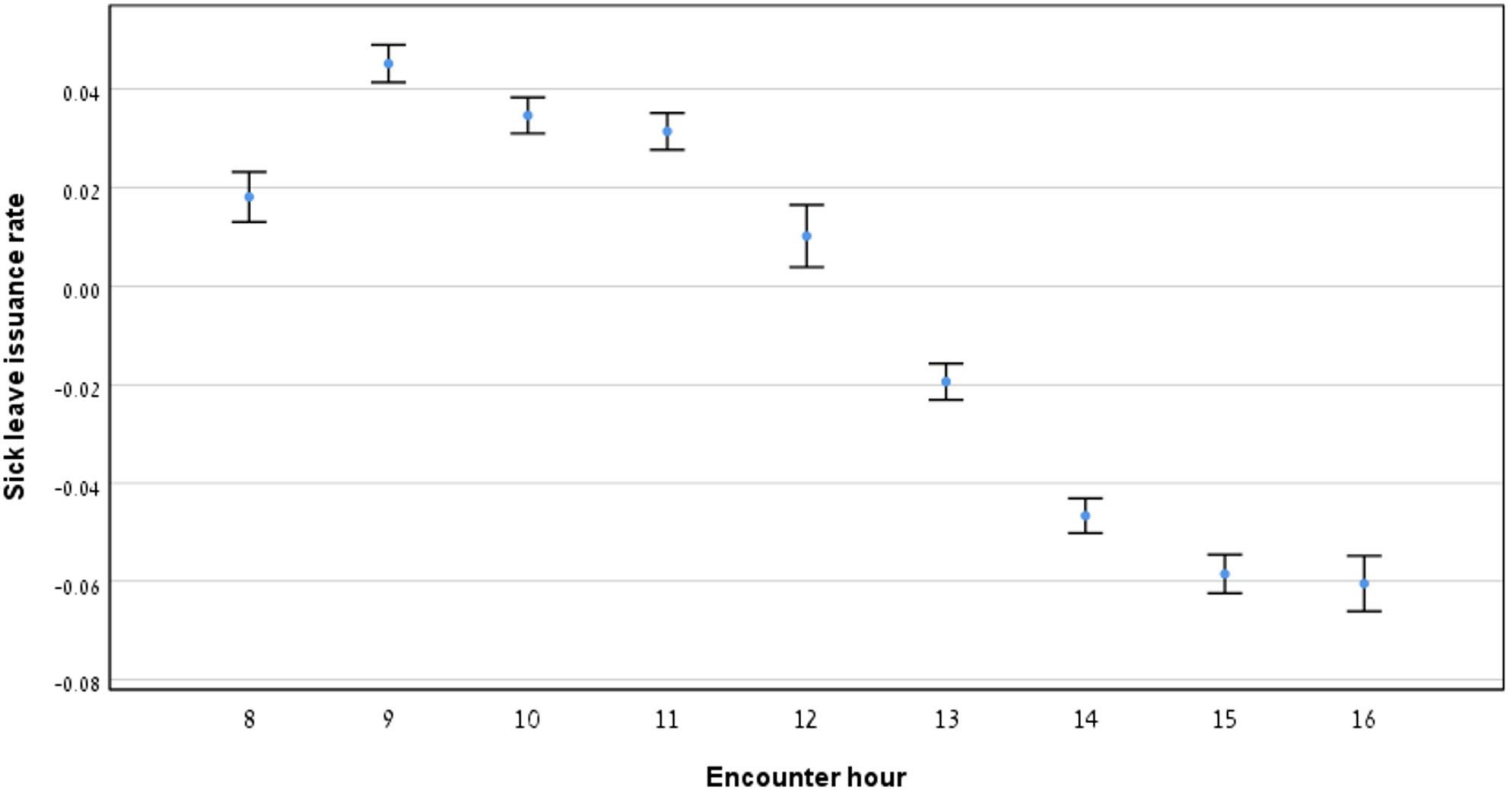
Sick leave issuance rates by encounter hour

Fig. 3 shows the proportion of encounters that resulted in sick leave being granted over the workday. Sick leave issuance remained relatively stable in the morning, rising slightly after the first hour, and then significantly decreased through the afternoon. Table 1 highlights encounter data across the morning, midday and afternoon hours, with statistically significant differences observed for comparisons across all time blocks (p < 0.05, Table 2).

### Relationship between average sick leave days granted and encounter hour

**Fig. 4:**
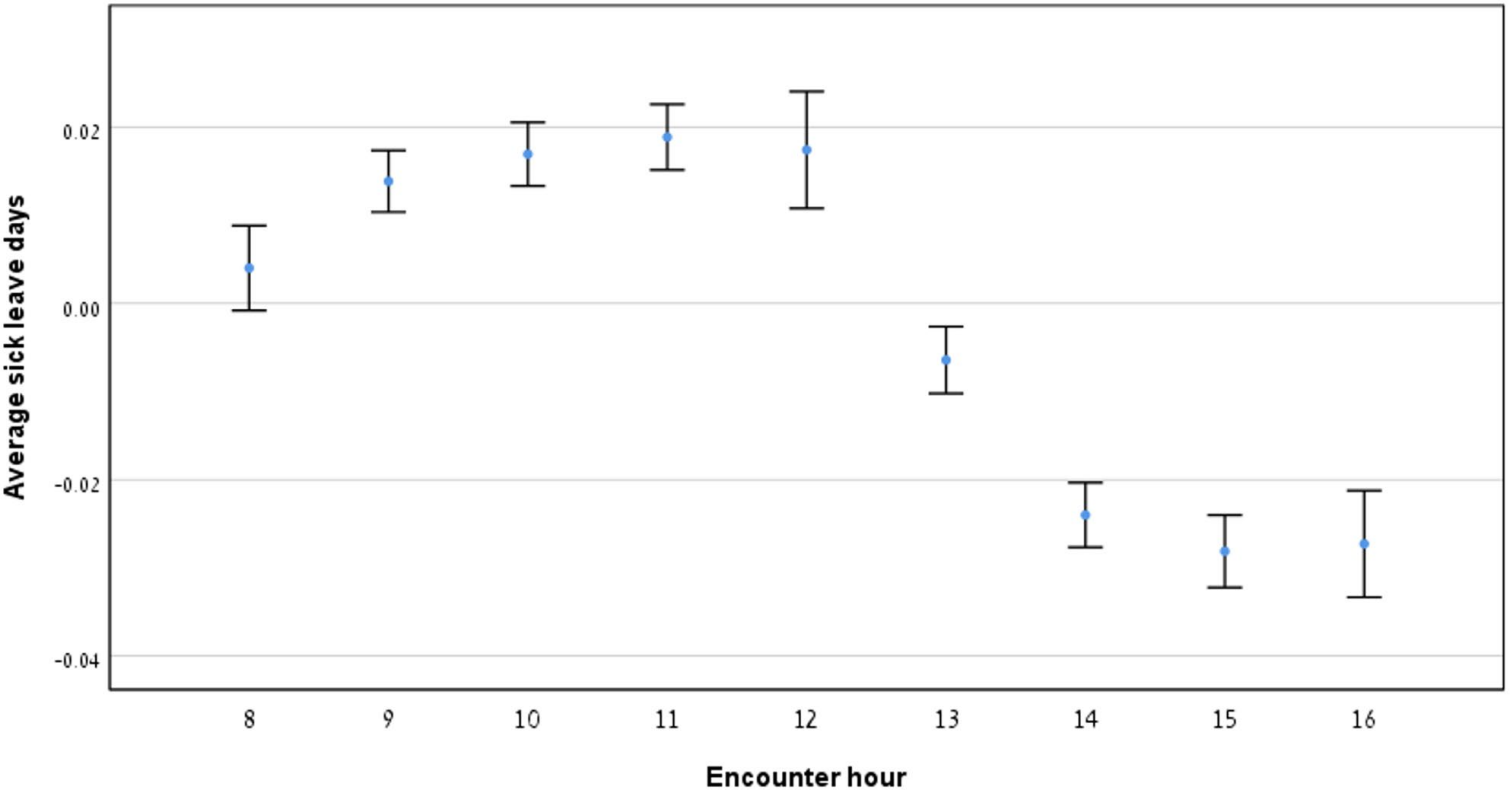
Average sick leave days granted by encounter hour

As illustrated in Fig. 4, the average number of sick leave days granted per encounter remained stable in the morning, increased approaching midday, and then significantly declined after the lunch break, continuing through the afternoon. Wilcoxon tests indicated significant differences (*p* < 0.05) across all time comparisons, confirming a downward trend in sick leave days granted over the course of the day.

### Relationship between provision of service exemptions and encounter hour

**Fig. 5:**
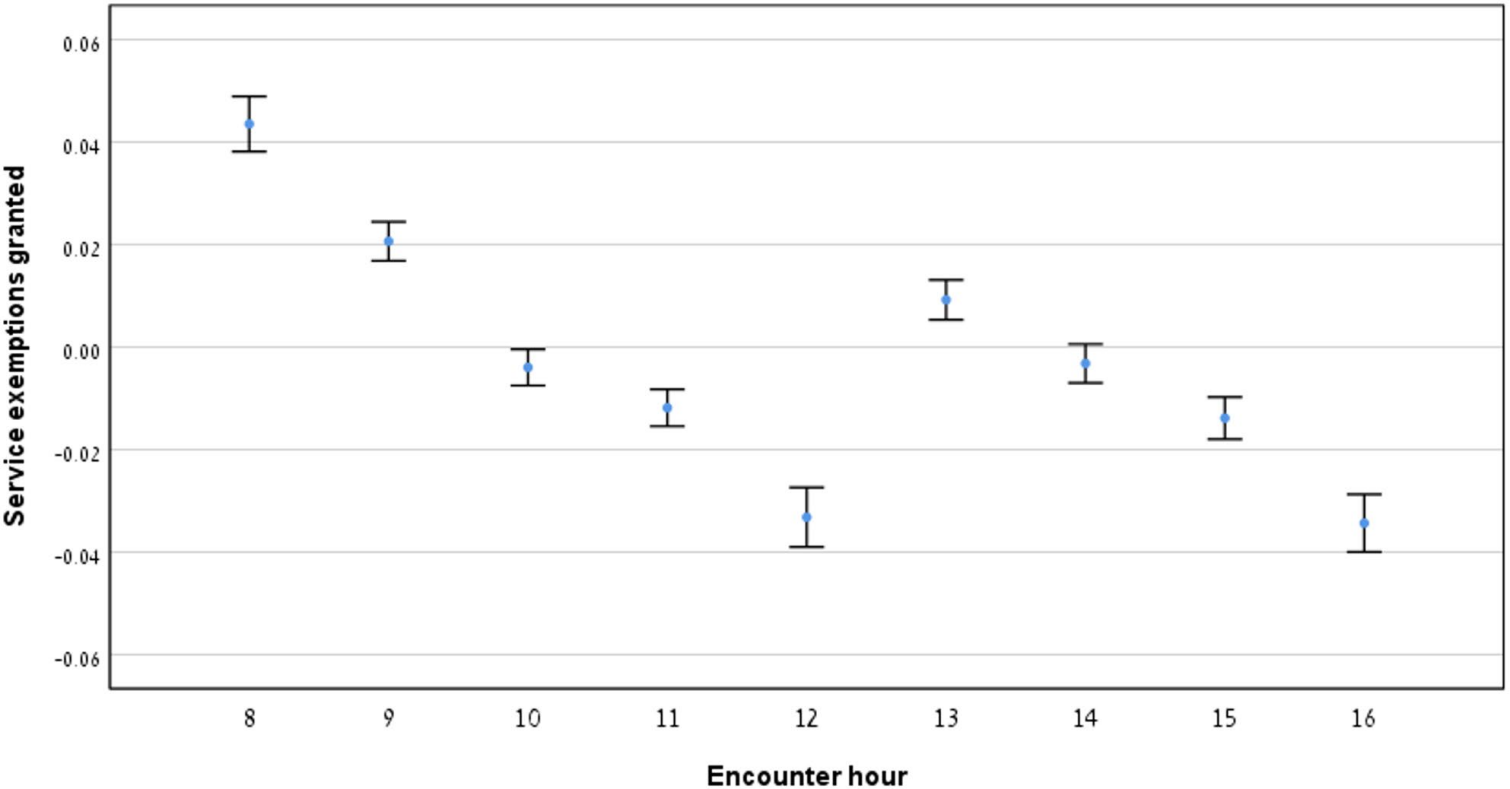
Service exemptions granted by encounter hour

Fig. 5 depicts the proportion of encounters resulting in service exemptions. A gradual decline was observed throughout the morning, followed by a brief increase post-lunch, before declining again in the late afternoon. Table 1 summarizes encounter data by morning, midday and afternoon hours. Statistically significant differences were observed across all time comparisons (*p* < 0.05, Table 2).

### Relationship between referrals for spinal X-rays for lower back pain and encounter hour

**Fig. 6:**
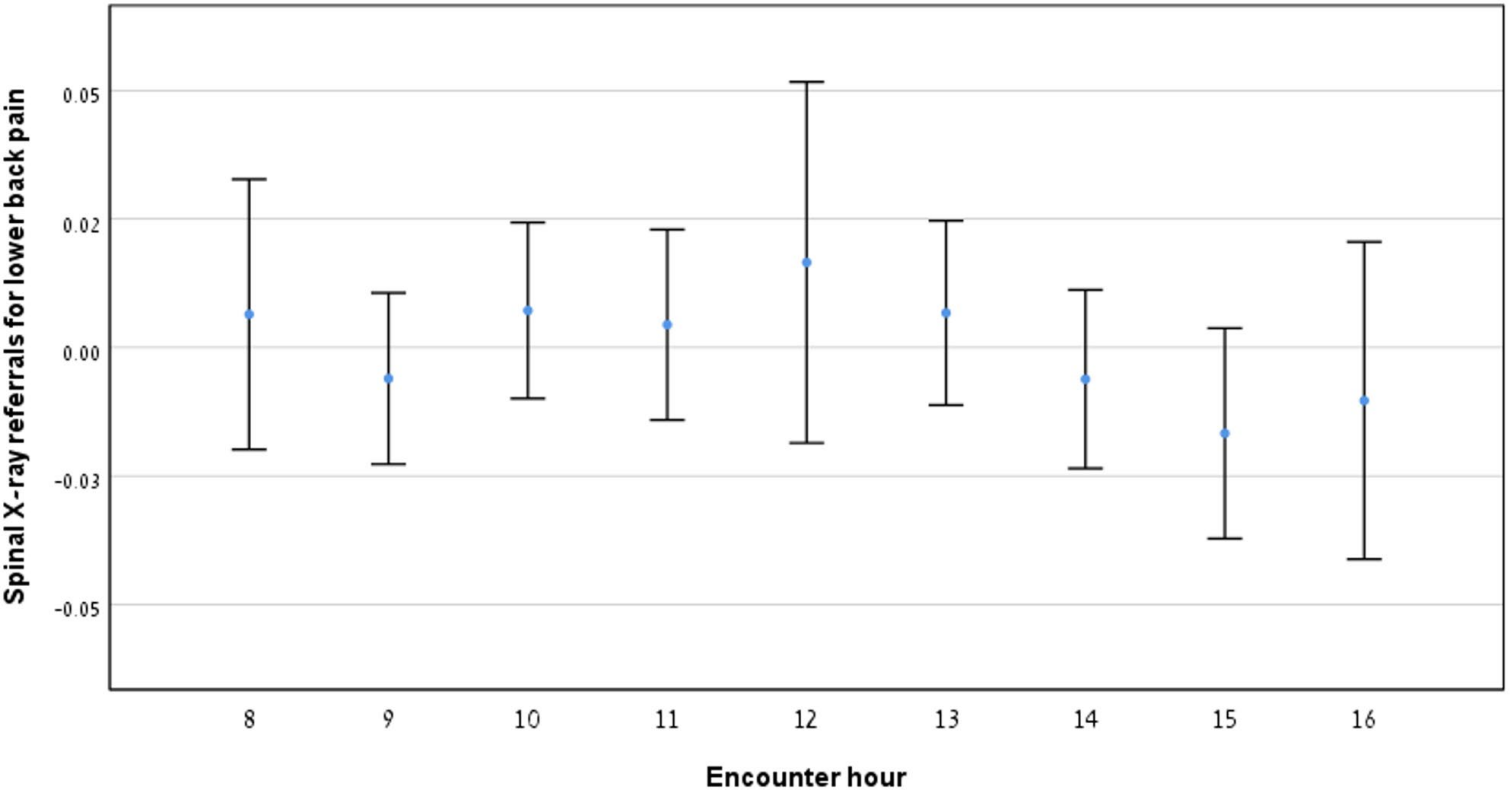
Spinal X-ray referrals for lower back pain by encounter hour

Fig. 6 shows the proportion of encounters for LBP that resulted in spinal X-ray referrals. No consistent trend was observed across the workday. Table 1 provides encounter data for morning, midday and afternoon hours. A statistically significant difference was found only between the hours of 13 and 16 (*p* < 0.05, Table 2), suggesting a slight decrease in referral rates during the afternoon.

### Relationship between antibiotic prescription for upper respiratory infections and encounter hour

**Fig. 7:**
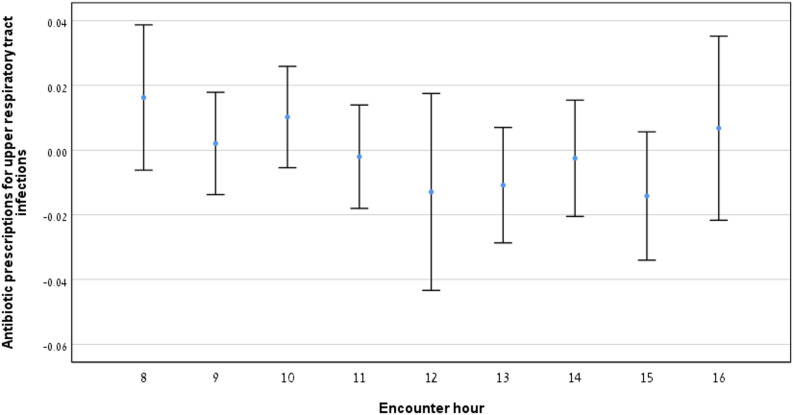
Antibiotic prescriptions for upper respiratory tract infections by encounter hour

Fig. 7 illustrates the rate of antibiotic prescriptions for URTI across working hours. No consistent trend was observed throughout the day. Table 1 presents encounter data for morning, midday and afternoon hours, with statistically significant differences found between 8 and 13, and 12 and 13 only (*p* < 0.05, Table 2).

### Effect of physician type on examined variables

Civilian physicians demonstrated a greater tendency to issue referrals, issue sick leave and provide service exemptions compared to their military counterparts. Spinal X-ray referrals for LBP, as well as antibiotic prescriptions for URTI, were also more common among civilian physicians. These differences were statistically significant across all examined variables (*p* < 0.05, Table 3). Notably, despite these differences, both military and civilian physicians exhibited similar trends in decision-making throughout the day.

**Table 3:** Relationship between physician type and their basic tendencies

Physician type	ER referrals % (95% CI)	Sick leave % (95% CI)	Service exemptions % (95% CI)	Spinal X-ray referrals for LBP % (95% CI)	Antibiotic prescriptions for URTI % (95% CI)
**Military physician**	2.3%	9.4%	5.7%	1.4%	7.4%
**Civilian physician**	2.6%	12.2%	7.2%	1.7%	9.6%
**p-value**	< 0.001	< 0.001	< 0.001	0.004	< 0.001

## Discussion

This retrospective analysis examined how encounter timing influenced various clinical outcomes among military physicians. Findings show that sick leave issuance and duration were highest in the morning and notably declined during the day. Granting of duty exemptions followed a bimodal trend, initially decreasing through the morning, peaking after lunch, and then dropping again by afternoon. Emergency referrals were less frequent in the morning, increased towards midday, dropped after lunch, and rose again towards day’s end. Unlike these variables, no discernable time-based trends were demonstrated for antibiotic prescriptions for URTI, or imaging referrals for LBP.

These results support the hypothesis that encounter timing may influence medical decisions, particularly for sick leave, duty exemptions, and emergency referrals, which appear sensitive to decision fatigue. Previous studies, such as Allan et al., have shown an increased likelihood for conservative choices, like emergency referrals, with accumulated decision fatigue [11]. Consistent with this, our data reflects a similar afternoon trend towards decisions that are effort-conserving and system-aligned, such as issuing fewer sick leaves and duty exemptions and more emergency referrals. In contrast to Linder et al., who observed an increase in antibiotic prescriptions later in the day attributed to decision fatigue [4], our data did not reveal a significant time-based pattern in antibiotic prescriptions for URTI, nor was there a significant association between time of day and LBP imaging referrals.

Decision fatigue may disproportionately affect military physicians, who navigate dual responsibilities: providing clinical care to soldiers while maintaining operational readiness. This dual role creates complex ethical and operational dilemmas, particularly when granting sick leave or duty exemptions—decisions that impact both individual soldiers’ well-being and unit functionality. Military physicians operate within a unique ethical and organizational framework, balancing beneficence and patient-centered care with systemic loyalty, military readiness, and command expectations. These competing forces—such as adherence to hierarchy, national duty, compassion, and professional autonomy—may shape decision-making, especially under conditions of fatigue. As cognitive and emotional resources are highest in the morning, physicians may initially be more willing to grant exemptions or sick leave, but as fatigue accumulates and ego-resources deplete, their inclination to make such resource-intensive decisions decreases. Under these circumstances, decision fatigue may heighten reliance on organizationally convenient, system-aligned choices, reducing cognitive and emotional strain. The physician’s individual risk perception, clinical autonomy, and moral compass may further mediate these tendencies. As noted by Lauor et al. [18], military medical personnel often face ethical dilemmas that intensify cognitive burden and may exacerbate decision fatigue. These underlying forces, while not directly measurable in this study, warrant further exploration in future research.

Civilian physicians tended to issue more sick leave and duty exemptions than their military counterparts, suggesting that military physicians’ stronger alignment with systemic objectives may influence their decision-making. Civilian physicians also displayed a higher propensity for antibiotic prescriptions and LBP imaging referrals, possibly reflecting differences in training or clinical approach. While outcome rates differed between civilian and military physicians, similar trends across the groups suggest shared susceptibility to decision fatigue.

The findings underscore decision fatigue as a possible factor influencing clinical practice, especially within military settings. Considering the scope of the data. These tendencies appear to be systemic and not strictly physician dependent, and thus may potentially indicate a structural vulnerability in clinical service delivery. If clinical decisions have predictable trends over the course of the workday, organizational workflow design and scheduling policies become important determinants of care consistency. Although this study did not directly measure health expenditures, the increase in emergency department referrals may contribute to downstream resource utilization, tertiary care burden and its associated cost. In a similar manner, rising exemption rates later in the day in the military setting affect operational readiness and unit-level functioning, extending the impact of decision fatigue beyond individual patient care to organizational performance.

Our study highlights the issue of decision fatigue in military medicine. Although civilian medicine differs in various characteristics, we believe that our study, as well as others in the medical literature, demonstrate that the phenomenon of decision fatigue seems to be broad. Structural pressures, such as high patient volume, time constraints and performance demands characterize many civilian public health systems as well, and support the potential generalizability of these findings, although further investigation is warranted, Future research should also prioritize healthcare utilization, downstream costs and system-level burden, as well as strategies to mitigate it.

Several limitations of this study should be noted. First, encounters were restricted to the hours of 8:00 to 17:00 and included only if the physician handled a minimum of 20 encounters in a day. Encounters outside these hours were excluded due to limited data, lower sample sizes and potential variance in patient urgency. This inclusion criterion ensured the analysis reflected decision-making among physicians with high clinical activity and minimized noise from sporadic providers. Future work may explore how decision fatigue manifests among low-volume or part-time physicians. Furthermore, we did not account for case severity mix across working hours, as the database does not support this variable. We also did not take into account physician workload across working hours as our database does not support it. This issue might influence clinical decisions [18] but its interactions with decision fatigue are not well studied. Additionally, lunch breaks were presumed to occur between 12:00 and 13:00, based on military organizational norms. While encounter frequency dropped significantly during this time, some variation in break timing may have occurred, and patient characteristics might also have differed around this time.

The dataset did not include variables such as day of the week, military branch affiliation or physician seniority, which could influence decision tendencies and risk perception. However, as described in Sect.  2.4, individual physician tendencies were partially accounted for through normalization, by standardizing each variable using physician-specific means and standard deviations. This approach aimed to minimize baseline variability between physicians, though future research may further explore additional contextual factors that could interact with fatigue-related trends.

Moreover, since direct measurement of decision fatigue is not feasible, several proxy indicators – emergency referrals, antibiotic prescriptions for URTI, LBP imaging referrals, sick leave issuance and service exemptions – were analyzed. While insightful, these proxies may not fully encompass decision fatigue’s complexity, necessitating cautious interpretation and further research.

## Conclusions

This study suggests that decision fatigue is a notable factor in clinical decision-making among military primary care physicians, as evidenced by time-based trends in granting sick leave, issuing duty exemptions, and recommending emergency referrals. These findings highlight the relevance of encounter timing in shaping physician decisions, especially in environments where clinical and organizational responsibilities intersect.

The observed trends reveal that decision fatigue may contribute to a gradual decline in the issuance of sick leave and duty exemptions as the day progresses, alongside an increase in emergency referrals. These patterns suggest a shift towards effort-conserving, system-aligned decisions under cognitive and emotional strain. The consistency of these trends across military and civilian physician groups underscores the pervasive impact of decision fatigue.

This study provides insight into how decision fatigue influences clinical practice. Increased awareness of its impact could improve decision-making processes and ultimately enhance the quality and consistency of care provided within healthcare systems. Addressing decision fatigue through strategies such as structured breaks, optimizing scheduling and workload management can help minimize its implications on patient care as well as healthcare utilization and expenditure. 

## Data Availability

The datasets analyzed during this study are not publicly available due to confidentiality restrictions related to military regulations. However, de-identified data may be available from the authors upon reasonable request and with appropriate permissions from the Israeli Defense Forces.

## Abbreviations

IDF: Israeli defense forces
CPR: Computerized patient record
LBP: Lower back pain
URTI: Upper respiratory tract infections
ER: Emergency room
